# Identification of antibody neutralization epitopes on the fusion protein of human metapneumovirus

**DOI:** 10.1099/vir.0.2008/005199-0

**Published:** 2008-12

**Authors:** Nancy D. Ulbrandt, Hong Ji, Nita K. Patel, Arnita S. Barnes, Susan Wilson, Peter A. Kiener, JoAnn Suzich, Michael P. McCarthy

**Affiliations:** MedImmune, Inc. 1 MedImmune Way, Gaithersburg, MD 20878, USA

## Abstract

Human metapneumovirus (hMPV) is genetically related to respiratory syncytial virus (RSV); both cause respiratory tract illnesses ranging from a mild cough to bronchiolitis and pneumonia. The F protein-directed monoclonal antibody (mAb) palivizumab has been shown to prevent severe lower respiratory tract RSV infection in animals and humans. We have previously reported on a panel of mAbs against the hMPV F protein that neutralize hMPV *in vitro* and, in two cases, *in vivo*. Here we describe the generation of hMPV mAb-resistant mutants (MARMs) to these neutralizing antibodies. Sequencing the F proteins of the hMPV MARMs identified several neutralizing epitopes. Interestingly, some of the epitopes mapped on the hMPV F protein coincide with homologous regions mapped previously on the RSV F protein, including the site against which the broadly protective mAb palivizumab is directed. This suggests that these homologous regions play important, conserved functions in both viruses.

Human metapneumonovirus (hMPV) is a recently described pathogenic respiratory paramyxovirus, with a disease pathology most similar to human respiratory syncytial virus (RSV). Both cause illness ranging from mild respiratory distress to bronchiolitis and pneumonia (van den Hoogen *et al.*, 2001; Williams *et al.*, 2006). RSV and hMPV have been subdivided into A and B subgroups based upon sequence diversity among isolates, and hMPV has been further dissected into sublineages denoted A1, A2, B1 and B2 (Peret *et al.*, 2002; van den Hoogen *et al.*, 2004). Like all paramyxoviruses, hMPV expresses a core group of genes, including three intrinsic membrane proteins: the fusion glycoprotein (F), attachment glycoprotein (G) and small hydrophobic protein (van den Hoogen *et al.*, 2002; Easton *et al.*, 2004). Viral coat proteins are prime targets for neutralizing antibodies and have been shown to elicit protective immunity in animal models. However, studies on the individual contributions of the hMPV coat proteins to the production of protective immunity showed that only the highly conserved F protein elicited a high-titre neutralizing antibody response (Skiadopoulos *et al.*, 2006). F protein immunization in varied formats induced protective immunity, in some instances protecting against subsequent challenge with a heterologous strain of hMPV (MacPhail *et al.*, 2004; Tang *et al.*, 2005; Ma *et al.*, 2005; Skiadopoulos *et al.*, 2004; Cseke *et al.*, 2007; Herfst *et al.*, 2007).

The ability of F-protein-directed monoclonal antibodies (mAbs) to neutralize RSV both *in vitro* and *in vivo* is well established (Johnson *et al.*, 1997; Wu *et al.*, 2005), perhaps best validated by the clinical use of the anti-RSV F mAb palivizumab as a prophylactic to reduce RSV disease in at-risk infants (Impact-RSV Study Group, 1998). The characteristics that make RSV F protein a good target for a broadly protective mAb are shared by hMPV F protein; we recently reported on a panel of 12 mAbs specific for hMPV F protein that effectively neutralized some or all hMPV sublineages either *in vitro* or *in vivo* (Ulbrandt *et al.*, 2006). These antibodies were classified into six groups based upon their ability to neutralize the four hMPV sublineage prototypes and to compete for binding to recombinant hMPV F protein.

To extend our knowledge of the hMPV F protein antigenic structure, mAb-resistant mutants (MARMs) were generated to nine of the 12 neutralizing mAbs reported by Ulbrandt *et al.* (2006). These mAbs spanned five of the six epitope groups that we had identified previously; we were unable to select MARMs against the group 1 mAb. To generate MARMs, virus isolates NL\1\00 (hMPV sublineage A1) or NL\1\99 (hMPV sublineage B1) at concentrations between 0.1×10^6^ and 5×10^6^ TCID_50_ were passaged in the presence of 50 times the IC_50_ of the antibodies in 24-well plates (the choice of virus sublineage was determined by its sensitivity to the mAb used for selection). For each mAb, 20–100 wells were scored for infection, in which 1–8 wells were positive for viral antigen production. Each individual positive well was passaged an additional two times in 50 times the IC_50_ of selection mAb. As hMPV does not form plaques or show substantial cytopathic effects in Vero cells, clonal isolation of the resistant mutants was not attempted with the expectation that individual positive wells would result from a limited number of viral particles. Following isolation, the viruses were retested for neutralization by the selection antibody, and in all cases they retained their resistance in a standard microneutralization assay (Ulbrandt *et al.*, 2006). Cross-neutralization of MARMs was performed as follows: each of the MARM virus preparations and the wild-type viral strains used to produce the MARMs (NL\1\00 and NL\1\99) was incubated with each of the antibodies (100, 10 or 1 μg ml^−1^). The MARM was considered resistant if it was not neutralized at a mAb concentration 10-fold above the IC_50_ for wild-type virus. The ability of the panel of mAbs to neutralize single isolates of the MARMs was then determined (Table 1). Much of the data from these experiments concur with the data collected previously from a competitive hMPV F protein binding ELISA (Ulbrandt *et al.*, 2006). For example, mAb 757, which did not compete with any other antibody in the ELISA, effectively neutralized all the MARMs isolated using the other antibodies and all other antibodies neutralized the mAb 757 MARM.

We next determined the sequences of the F protein gene for all the isolated MARMs. RNA was extracted from infected cells using the RNAeasy kit (Qiagen). cDNA was made using random primers with the You-Prime-It cDNA kit (GE Healthcare). The cDNA was used in a PCR to isolate the soluble portion of the F protein using the following primers: NL\1\00 5′ primer (5′-GGATCCCCTTAAAGAGAGCTACTTAGAAGAG-3′) and 3′ primer (5′-GAATTCTTAGCCAGTGTTTCCTTTCTCTGC-3′) or NL\1\99 5′ primer (5′-GGATCCCCTAAAGGAGAGTTATTTGGAAG-3′) and the NL\1\00 3′ primer. PCR products were sequenced using ABI BigDye dye terminator v3.1 and were analysed on an ABI DNA analyser.

The sequences of the F protein were compared to the sequences of the input virus used for selection, as well as to virus passaged in the presence of an irrelevant mAb. In many cases, the same mutations were found in multiple isolates; however, each individual mutation is shown only once in Table 2. MARMs used for the cross-neutralization studies are italicized and underlined. Epitope group 2 MARMs derived from mAb 757 were sequenced and found to have mutations at aa 132 and 152 (Table 2). This region is in the first heptad repeat of the hMPV F protein (a schematic of the domain structure and MARM mutation sites of hMPV and RSV F protein is provided in Fig. 1) and although the mutations are not close in primary sequence, this region is predicted to form an extended *α*-helix (post-fusion) and aa 132 and 152 might present on the same face of this helix.

F protein mutations in MARMs generated against the epitope group 3 antibody pair, mAbs 967 and 1025, map to a region between aa 177 and 179 (Table 2). Aa position 179 is one (of only three) that discriminates the B1 F protein sequence from all other hMPV sublineages; arginine in the B1 prototype NL\1\99, lysine in all others. The group 3 mAbs have no neutralizing efficacy against NL\1\99, and NL\1\00 MARMs generated against the group 3 mAbs contained a lysine to arginine change at this position. The other mutations at this position resulted in either a change in charge or the addition of a bulkier side group, suggesting that aa 179 makes direct contact with a complementarity-determining region on the group 3 mAbs; the mutation at position 178 may alter how aa 179 is disposed in the folded structure.

The epitope group 4 antibodies, of which mAbs 234 and 338 have been shown to protect against challenge with both A1 and B1 sublineages of hMPV *in vivo*, required high levels of virus in order to isolate a small number of escape mutants. The MARMs derived from selection with mAbs 338 and 234 were resistant to all three antibodies in group 4, but MARMs selected against mAb 628 selection were only resistant to mAb 628 (Table 1). Sequence analysis of the MARMs was consistent with the results of the neutralization assays, in that all three antibodies selected for mutations in the same region of the F protein but the mutations isolated by selection with mAb 628 were different to the mutations found in the mAb 338 and 234 MARMs. This suggests that the binding site of mAb 628 is different but adjacent to the mAb 234 and 338 binding sites, in agreement with competition ELISA results.

The epitope group 5 and 6 MARMs were sequenced and mutations were found to map to position 386–397 of the hMPV F protein (Table 2). The location of these mutations confirms that mAbs 710 and 836 bind to the same region. The mutations in the F protein isolated by selection with mAb 659 were proximal to the mAb 710 and 836 mutations but did not confer resistance to all of the other antibodies in these groups (Table 1). In addition, the region recognized by these antibodies contains amino acid differences between the A1, A2 and B2 prototypes at position 396, providing (as above for the group 3 mAbs) a possible explanation for the different sensitivities of the hMPV sublineages to the group 5 and 6 mAbs.

The characterization of resistant mutants is one of the primary tools of virus structure/function research. Our work on hMPV, both in developing neutralizing antibodies and in generating MARMs, has been informed by the instructive experience of ourselves and others with RSV. The antigenic structure of RSV F protein has been defined in a number of ways. First, panels of antibodies were generated against RSV F protein and these were classified into different groups based on their ability to bind different RSV strains or to compete for binding to the same strain (e.g. Beeler & van Wyke Coelingh, 1989). The physical antibody binding sites on the RSV F protein were primarily determined in two ways. In some cases, the antibodies bound to peptide segments of the target antigen (e.g. Lopez *et al.*, 1993; Lounsbach *et al.*, 1993; Langedijk *et al.*, 1998) or MARMs were selected and the locations of the amino acid changes on the RSV F protein that conferred resistance were identified (e.g. Arbiza *et al.*, 1992; Crowe *et al.*, 1998; Lopez *et al.*, 1998; Zhao *et al.*, 2004). The combination of the competition, cross-neutralization/binding, peptide binding and MARM mutational data has provided a fairly detailed picture of the functional domain architecture of the RSV F protein.

A schematic depiction of RSV F protein domain structure is illustrated on the lower half of Fig. 1, with the approximate location of the primary immunogenic sites along the linear sequence indicated. The A site was first delineated by Beeler & van Wyke Coelingh (1989) and the antigenic areas I–VI were classified by Melero and co-workers (Arbiza *et al.*, 1992; Lopez *et al.*, 1998). The homologous domain structure of the hMPV F protein is indicated on the upper half of Fig. 1; the alignment of hMPV and RSV F protein structures is based both on amino acid sequence comparison (which only averages ∼33 % sequence identity between hMPV and RSV subgroups) and on the predicted locations and boundaries of functional domains. The relative location of the amino acid changes associated with the MARMs that were selected by our panel of anti-hMPV F protein mAbs indicate that some of these mutational hotspots share common locations on both RSV and hMPV F protein.

The epitope group 2 MARMs localize to two amino acid positions (132 and 152) within the predicted heptad repeat 1 of hMPV F protein. No RSV MARMs that localize to this region have been reported, but a neutralizing mAb generated against bovine RSV F protein has been suggested to bind to this region (Langedijk *et al.*, 1998). Similarly, no RSV MARMs have been mapped to the region homologous to epitope group 3 on hMPV F protein, but neutralizing mAbs have been generated against a peptide that spans a corresponding region of RSV F protein (Corvaisier *et al.*, 1997). Finally, epitope groups 5 and 6 map to a neutralizing antigenic region on hMPV F protein which overlaps antigenic region IV and is slightly N-terminal to the antigenic regions V and VI on RSV F protein (Arbiza *et al.*, 1992; Lopez *et al.*, 1998).

From a clinical perspective, the epitope group 4 mAbs 234 and 338 are the most interesting, as these mAbs neutralize prototypic hMPV A1 and B1 viruses both *in vitro* and *in vivo* (Ulbrandt *et al.*, 2006). Of note, the site that these mAbs recognize on the hMPV F protein corresponds to the cognate A site or site II defined for RSV F protein (Beeler & van Wyke Coelingh, 1989; Arbiza *et al.*, 1992) that is recognized by the neutralizing anti-RSV monoclonal palivizumab, which is effective at reducing RSV disease in humans (Impact-RSV Study Group, 1998). mAbs to epitope 4 of hMPV F protein target the most conserved epitope found in all sublineages of hMPV. As with RSV, this region probably plays an important role in the virus and may only tolerate minor changes. Based on the experience with palivizumab and RSV disease, this suggests that mAbs to this region in hMPV F protein could have clinical potential.

The mechanism by which F protein-directed mAbs neutralize virus (either hMPV or RSV) is still unresolved. Steric blockage may be involved, but a more likely mechanism of action would involve binding to a ‘pre-fusion’ conformation of the F protein and inhibiting the hairpin formation between the first and second heptad repeats currently modelled to bring the viral and target cell membranes into apposition and subsequent fusion (Zhao *et al.*, 2000; Lamb *et al.*, 2006; Miller *et al.*, 2007). These models suggest that mAb neutralization could involve binding to sites in the F protein important to this conformational transition. These could be binding either initially distal sites which must come into proximity or regions which serve as a hinge, or simply by stabilizing the pre-fusion conformation in some way. As previously reported (Ulbrandt *et al.*, 2006), the epitope group 6 mAbs compete for binding with the epitope group 4 mAbs, even though the mutations associated with their particular MARMs are ∼150 aa apart in the primary sequence. This suggests that these epitopes are adjacent in the folded three-dimensional structure, in agreement with homology modelling of the hMPV F protein based on the structures of Newcastle disease virus (Smith *et al.*, 2002) and human parainfluenza virus (Morton *et al.*, 2003) F proteins.

A final point of note is the low number of broadly neutralizing mAbs we derived that are directed against hMPV F protein. Due to the high degree of conservation of the F protein, it is somewhat surprising that more of the neutralizing antibodies were not pan-specific. Throughout the extra-membranous region of the hMPV F protein (roughly 450 aa in length) there are only 25 positions that vary within and between sublineages. The observation that variations occur in only ∼6 % of the amino acids in the extra-membranous region of hMPV suggests that most of the amino acid positions in the F protein are crucial to its function. In conclusion, our studies emphasize the structural and functional similarities of the fusion proteins of RSV and hMPV.

## Figures and Tables

**Fig. 1.**
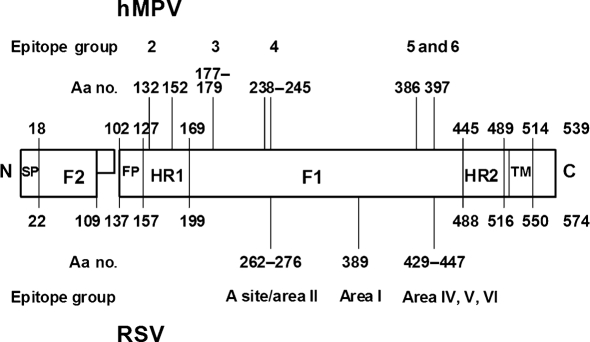
Schematic depiction of hMPV and RSV F protein domain structures and relative location of MARM mutation sites. Indicated are the N terminus (N), signal peptide (SP), fusion peptide (FP), heptad repeat 1 (HR1), heptad repeat 2 (HR2), transmembrane domain (TM) and C terminus (C), as well as the F1 and F2 segments of the F protein. The amino acid positions that border domains (predicted) or cleavage sites and C termini (known) are indicated. Also depicted are the relative positions of the hMPV epitope group MARM mutations and corresponding MARM mutation sites on the RSV F protein (see text for details).

**Table 1.** Cross-neutralization analysis of MARMs R, indicates the MARMs that were resistant to neutralization with the antibody listed. MARMs were not generated against mAbs 242 and 344.

**Antibody tested**		**Antibody used for selection**								
		**Group 2**	**Group 3**		**Group 4**			**Group 5**		**Group 6**
		**mAb 757**	**mAb 967**	**mAb 1025**	**mAb 234**	**mAb 338**	**mAb 628**	**mAb 659**	**mAb 710**	**mAb 836**
**Group 2**	mAb 757	**R**								
**Group 3**	mAb 967		**R**	**R**						
	mAb 1025		**R**	**R**						
**Group 4**	mAb 234				**R**	**R**				
	mAb 338				**R**	**R**				
	mAb 628				**R**	**R**	**R**			
**Group 5**	mAb 344								**R**	**R**
	mAb 659							**R**		
	mAb 710								**R**	**R**
**Group 6**	mAb 242								**R**	**R**
	mAb 836								**R**	**R**

**Table 2.** Amino acid variations in hMPV MARMs The italicized mutations are those that were used in generating the cross-neutralization data in Table 1.

**Strain**	**Selection**	**Epitope group**	**Aa**											
			**132**	**152**	**177**	**178**	**179**	**238**	**239**	**241**	**242**	**245**	**386**	**397**
NL\1\99	Control IgG		**S**	**G**	**I**	**N**	**R**	**A**	**G**	**I**	**K**	**L**	**K**	**V**
MARMs	mAb 757	2	***R***											
				**R**										
	mAb 659	5/6											***E***	
	mAb 710	5/6												***F***
	mAb 836	5/6												***F***
NL\1\00	Control IgG		**S**	**G**	**I**	**N**	**K**	**A**	**G**	**I**	**K**	**L**	**K**	**W**
MARMs	mAb 967	3					***E***							
							**N**							
							**R**							
	mAb 1025	3				***Y***								
							**E**							
					**V**		**R**							
	mAb 234	4									***N***			
	mAb 338	4								**R**				
											***N***			
								**E**			**N**			
								**T**			**N**			
								**E**			**T**			
	mAb 628	4						**E**				**S**		
									***E***			***S***		
								**E**						
									**E**					
